# Deconstructing the Self-Other Binary in Care Networks by Unravelling Professional’ Perspectives through an Intersectional Lens

**DOI:** 10.1007/s10728-024-00504-z

**Published:** 2025-01-03

**Authors:** Aldiene Henrieke Hengelaar, Margo van Hartingsveldt, Tineke Abma, Petra Verdonk

**Affiliations:** 1 Department Ethics, Law and Humanities, Amsterdam Public Health Research Institute, Amsterdam University of Applied Science, Amsterdam UMC, location VUmc, Tafelbergweg 51, PO box 2557, 1000 CN Amsterdam, The Netherlands; 2 Department of Occupational Therapy, Amsterdam University of Applied Science, Amsterdam, The Netherlands; 3 Department Public Health and Primary Care, Leiden University Medical Centre, Leiden University and Leyden Academy on Vitality and Ageing, Leiden, The Netherlands

**Keywords:** Intersectionality, Care networks, Informal carers, Collaboration, Acquired brain injury

## Abstract

In many western countries informal care is conceived as the answer to the increasing care demand. Little is known how formal and informal caregivers collaborate in the context of an diverse ageing population. The aim of this study was to gain insight in how professionals’ perspectives regarding the collaboration with informal carers with a migration background are framed and shaped by intersecting aspects of diversity. We used an intersectionality informed qualitative design with informal conversations (N = 12) and semi-structured interviews (N = 17) with healthcare professionals working with clients with Acquired Brain Injury. Two critical friends were involved in the analysis which was substantiated by a participatory analysis with a community of practice. We identified four interrelated themes: (a) ‘*The difficult Other*’ in which professionals reflected on carers with a migration background causing ‘difficulties’; (b) ‘*The dependent Other*’ refers to professionals’ realization that ‘difficulties’ are intensified by the context in which care takes place; (c) in ‘*The uncomfortable self*’ professionals describe how feelings of insecurities evoked by the Other are associated with an inability to act ‘professionally’, and; (d) ‘*The reflexive self*’ shows how some professionals reflect on their own identities and identify their blind spots in collaboration within a care network. These themes demonstrate the tensions, biases and power imbalances between carers and professionals, which may explain some of the existing health disparities perpetuated through care networks.

## Introduction

In many western countries the informal carers are considered essential to face the increasing demand of care in an ageing population [33]. Informal care refers to carers who in contrast to professional- or formal carers belong to the existing social network of the care recipient, e.g. family, friends and/or neighbors, and provide unpaid care to someone with long-term health and/or welfare needs. The context of this study is the Netherlands, where the numbers of carers has risen to 5.5 million in 2019 due to the changes in health policies by strengthening the focus on the self-reliance of citizens and their social networks ([12], p.7). Informal care often occurs within larger care networks in which collaboration with different professionals is necessary, referring to care in the home context as well as within institutional settings. In the Netherlands, carers often reside in the inmost layer of these care networks together with the care recipient. Due to rising healthcare costs, an aging society, and austerity measures within the Dutch healthcare system, there is a growing need for informal care [16]. Even though the provision of informal care can be a positive experience for carers, the hyper-focus on informal care often ignores carers’ potential overload and pressure on their labor participation as well as safety risks and lower standards of care [12] currently, one out of ten feels overburdened [9].

The existing literature on the collaboration between professionals and informal carers in diverse networks shows that a ‘partnership approach’ contributes to quality care within care networks and to the wellbeing of informal carers [29]. This requires an open dialogue between all actors, about the mutual understanding of expectations and assumptions regarding collaboration, roles and needs. However, such partnerships can be difficult to achieve in practice due to the complex contexts surrounding the collaboration and a lack of awareness about the role that underlying intersecting aspects of diversity play in shaping expectations and assumptions within care networks [31]. Aspects of diversity such as gender, age, ability, religion, culture, geography, ethnicity, education level, socioeconomic status simultaneously and in interaction shape everyday experiences and further complicate the collaboration between professionals and informal carers in diverse care networks [18].

In this study, we focused on professionals’ experiences of collaborating with carers who migrated to the Netherlands and act as a proxy for their care recipients with acquired brain injury. Migration to the Netherlands has been shaped by the country’s colonial history, economic factors such as labor migration, as well as by political choices and public policies [36]. Carers with a migration background cannot be categorized as one homogeneous group and multiple within group difference exist—their backgrounds may be European or from other continents with differing ethnicities, educational levels may vary as well as for example religious affiliation or income level. They do share higher risks of discrimination in society as well as within health care settings. Our earlier study showed that carers experienced a sense of injustice after experiences of discrimination, leading to unmet needs of care recipients and carers themselves, and a loss of trust in healthcare professionals [30]. Additionally when zooming in on this collaboration it can be seen that carers with a migration background report lower levels of wellbeing when they are misunderstood within care situations or when they encounter racism within care networks [40]. According to Shepherd et al. [49] different studies in the field of public health indicate that particular cultural groups are often underserved, experience negative treatment and/or treatment outcomes (p. 2). There is a need to further understand the contextual nature of this process [41], as professionals’ perspective on their collaboration with carers with a migration background is largely understudied [31].

There are indications that these negative experiences and outcomes of the care for people with a migration background and informal carers are related to the context and social position of professionals. Claeys et al. [19] show, for instance, that professionals take their own frame of reference as a starting point during care planning and often encounter difficulties empathizing with other frames of reference. This might stem from the fact that professionals sometimes experience dealing with diversity as stressful [17] and often find it easier to provide care to someone who has a similar background [43]. Berdai Chaouni [10] adds that professionals caring for people with a migration background appear to do so in a rather ethnocentric and essentialist context and are often unaware of the complex reality of care. This results in increased informal care burden and might contribute to ineffective care in care networks (p.8). This phenomenon can be explained by professionals’ positionality in that specific care situation, showing the impact of personal and professional identities in a specific situation, based on for example age, gender, and professional training [54]. An analysis of positionality can show how personal and professional identities influence and potentially bias or strengthen their understanding of the collaboration [44] and provide insight in how unconscious bias might create unfair systems of power in care networks [40].

This study aims to explore diverse professional perspectives on and experiences in collaboration with carers with a migration background in care networks around care recipients with acquired brain injury ABI in order to expose aspects of power and injustice in care networks. We deliberately chose to focus on professionals working in care networks around a care recipient with acquired brain injury (ABI)—the leading cause of disability worldwide [48]—because care recipients with ABI require long-term complex care, provided at home where care networks often consist of different professionals [4]. This study is part of a larger research project where the experiences with the collaboration in care networks is central and the perspectives of professionals, informal carers and care recipients are explored. The stories of informal carers and care recipients are of equal importance and will be featured in two different studies. We focus here on the perspectives of professionals. Our study builds further on the notion that the social position of professionals influences the collaboration with informal carers, and aims to explore the experiences of professionals in ‘diverse’ care networks by explicitly paying attention to how systems of power play a role in these care networks [6].

## Theoretical Background

To understand professionals’ perspectives within diverse care networks, it is essential to gain insight in how their perspectives are framed and shaped by intersecting aspects of diversity as well as situational and contextual factors [54]. Within public health there is a longstanding commitment to understand how individual and structural identities, characteristics and patterns shape health and wellbeing [38]. Social categories and structures as well as individual identities create the conditions in which people live and influence their health and well-being [45]. An intersectionality approach, originally coined by black feminist legal scholar Kimberley Crenshaw, may offer insight in the interactions between the various aspects of diversity and (dis)advantage in individual lives, social practices, institutional arrangements, cultural ideologies and the outcomes of these interactions in terms of power, social inequities and health disparities [27, 28]. This study does not solely focus on cultural aspects or differences, but goes a step further to explore the influence of the multiple intersecting aspects of diversity in the context of care networks. The intersectionality lens helps to embrace rather than obscure the heterogeneity of peoples lived experiences [13] and serves as a critical praxis focusing on (in)equality and social (in)justice in care networks [38].

By doing so, intersectionality is understood as challenging health inequalities and structural disadvantages within care networks [31, 38]. Inequalities are not merely differences but unfair disparities, and might stem from the process of Othering in which people who are constructed as deviating from a fictitious societal norm are identified as being different, creating an us versus them mentality [14]. Othering is a process that ascribes inferior characteristics to minority groups and to people deviating from social norms in order to establish and ideology of superiority of majority groups. Othering is identified by Claeys et al. [19] as a key concept underlying difficult communication and potential conflicts within care networks, especially when care is provided to care recipients with an ethnic minority background. Leyerzapf [35] shows that the denial of underlying in- and exclusion mechanisms within Dutch health care are linked to white innocence [53] and white privilege [21]. An analysis of processes of Othering requires a critical examination the taken for granted social position of privilege of professionals [51, 55].

## Methodology

In this study an intersectionality informed qualitative design was used [25, 27, 32]. From an epistemological standpoint we recognize that power is relational which shapes perceptions as well as presuppositions which are context based and incorporate time contingency [26, 39]. Using intersectionality as a way of thinking recognizes the interconnectedness of numerous socially constructed identities which collectively shape the lived experiences of individuals and groups [3]. Additionally, since the larger research project follows is a Participatory Action Research (PAR), we have taken measures to ensure the inclusion of stakeholders in every step of the research process [1], which stems from the epistemological belief that knowledge is embedded in social relationships and is most influential when produced collaboratively [22].

### Data Collection

**Fig. 1:**
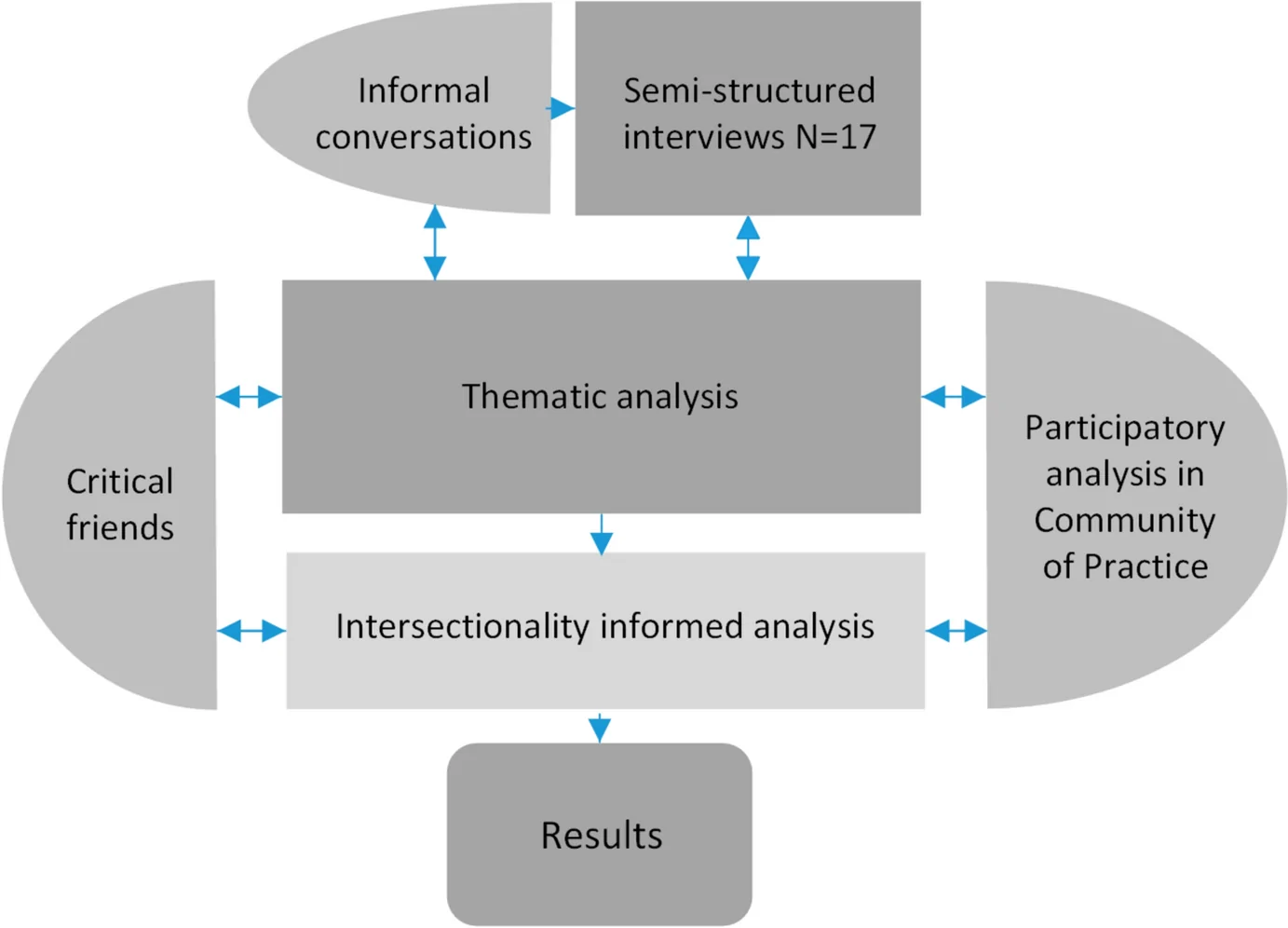
visualization of data collection and analysis

Two methods of data collection were used in order to elicit rich data from different perspectives, to ensure rigor and establish trustworthiness. These are visualized in Fig. 1. Firstly, informal conversations were conducted with professionals in twelve health and/or welfare organizations who support carers. These professionals were social workers, community nurses or community coaches. The purpose of these informal conversations was to: (a) recruit participants for this and other sub-studies; (b) use their expertise to inform the interview guide for the additional interviews, and: (c) substantiate the data analysis and strengthen our understanding and interpretation. Secondly, seventeen semi-structured interviews were conducted with various healthcare professionals.

Everyday experiences of professionals in care networks with carers with a migration background were central in the interview guide and open questions were used, example questions from the interview guides are shown in Table 1. Questions that frame social categories as binary or provoke an essentialized understanding of said categories were avoided [13].

**Table 1:** example questions interview guide

Can you describe your experiences in collaborating with carers with a migration background?
How are the care responsibilities arranged?
What goes well and what needs to be improved?
What does it mean for you to collaborate with carers with a migration background?
What is important for you in the collaboration with carers with a migration background?

### Sample

Sampling was done purposively to include a diverse set of healthcare professionals working with carers with a background of migration [23]. From an intersectional perspective this allows thinking beyond existing categories of difference [32], p.8), meaning that we did not start with predetermined categories of difference within the group healthcare professionals working with carers with a background of migration. As a starting point for sampling we focused on majority Dutch professionals working with carers with a migration background Recruitment was done via email, telephone and via informal conversations with key figures in healthcare- and carer support organizations, starting with the existing network of the authors after which snowball sampling was used [23]. For this study, we were particularly interested in the perspective of Dutch majority professionals. During informal conversations within care organizations it became clear that professionals with a background of migration themselves wanting to share their experiences. They argued that too often they collaborate in care networks with carers with a migration background that is not always similar to their own background. These collaborative networks provided positive and complex experiences they wished to share, as eloquently described by Ahmad [7]. Therefore, we did invite a diverse group of professionals along-the-way (emergent sampling) [23, 25]. A description of the characteristics that were identified by the participants as relevant during the interviews is presented in Table 2.

**Table 2:** Intersectional visualization of sample

Participant	Profession	Place of Residence	Ethnicity	Age	Place of work	Gender
I1	Policy Officer	Urban	Dutch	63	Municipality	Women
I2	Policy Officer	Urban	Dutch	52	Municipality	Women
I3	Policy Officer	Urban	Dutch	34	Policy Advisory organization on informal care	Men
I4	Community Nurse	Urban	Dutch-Peruvean	36	Care organization also providing home care	Women
I5	Community Nurse	Urban	Dutch-Surinam	53	Care organization also providing home care	Women
I6	Community Nurse	Urban	Dutch	46	Care organization also providing home care	Women
I7	Occupational Therapist	Urban	Dutch	25	Rehabilitation Centre	Women
I8	Occupational Therapist	Urban	Dutch-Armenian	48	Rehabilitation Centre	Women
I9	Occupational Therapist	Urban	Dutch-Turkish	41	Nursing Home	Women
I10	Occupational Therapist	Urban	Dutch	32	Nursing Home	Women
I11	Occupational Therapist	Urban	Dutch	27	Care organization also providing home care	Women
I12	Occupational Therapist	Urban	Dutch	46	Primary care practice	Men
I13	Occupational Therapist	Urban	Dutch	38	Primary care practice	Men
I14	Creative Therapist	Urban	Dutch-Surinam	42	Care organization also providing home care	Women
I15	Social Worker	Urban	Dutch-Afghan	36	Policy Advisory organization on informal care	Women
I16	Social Worker	Urban	Dutch	47	Rehabilitation Centre	Women
I17	Director	Urban	Dutch	55	Care organization also providing home care	Men

### Analysis

Analysis consisted of a thematic analysis [20] and a secondary intersectionality informed analysis [50]. Researcher triangulation was a priority to ensure rigor and establish trustworthiness, therefore the analysis was substantiated by the involvement of two critical friends and a participatory analysis in a community of practice (CoP) (see Fig. 1). Two critical friends were involved during the preliminary analysis [11], both of them identified as bicultural, and provided different perspectives as they were able to look from a distance to the data and the analysis, yet also providing additional insights in what it means to have a migration background.

Interview transcripts and fieldnotes from informal conversations were, in constant comparison, subject to a thematic analysis, following the steps of Clarke & Braun [20]. Subsequently, a round of intersectionality-based analysis was performed, which allows for analyzing complexity, the interaction between individual and institutional levels as well as focusing on power structures and social injustices. Based on Stuij et al. [50], Hunting [32] and McCall [37], an intersectional informed coding scheme was developed (see Table 3). This intersectional analysis deepens the thematic analysis by zooming in on the social locations participants assign to themselves [50]. Within the themes, identified in the thematic analysis, we: (a) searched for social categories participants assigned to themselves; (b) analyzed which intersections of social categories came forward as relevant within the data; (c) focused on processes of social injustice and power structures within the stories of the participants, and finally; (d) searched for aspects of relevance mentioned with regard to the socio-cultural, historical and/ or political context in which the provided care took place, in order to expose aspects of power and injustice in care networks.

**Table 3:** intersectionality informed coding scheme (Stuij, et al. [50]; [32, 37])

(1) Which social categories do the participants assign to themselves?	e.g. gender, age, disability, family relations, ethnicity, religion, urban/rural, SES, occupation, education level
(2) Which intersections of social categories come forward as relevant within the data?	linking social categories to the stories told by the participants
(3) Which aspects of social and societal injustice come forward in the data? Which forms of power, power structures, power inequities detected in the interview data?	This can be examples from both justice and injustice What is used as the norm? Which assumptions, prejudice, norms and values come forward? How does the collaboration take place and can tensions be detected?
(4) Which aspects of the socio-cultural, historical and or political context come forward?	Locally, nationally and of in the country of origin? Which assumptions, prejudice, norms and values come forward?

A Community of Practice (CoP), running parallel to the larger research study, was involved in additional participatory analysis aiming to ensure input from representatives that are impacted by this study [2]. The CoP consist of care recipients with an ABI, carers and different professionals (N = 8) with their engagement in the analysis, the results are deepened and strengthened by their perspectives with respect to all forms of knowledge to ensure that dominant logics that uphold inequalities will not be reproduced [24]. Five meetings were held with the CoP guided by creative hermeneutics [15]: (1) a group discussion of relevant themes from the thematic analysis; (2) an unraveling of our own pre-assumptions towards the topics; (3) reflections on the CoP members positions in everyday life; (4) a group discussion about the CoP members perspective on collaboration in care networks, what do they recognize in their daily practice and; (5) mind-mapping the connection between the thematic and intersectional analysis in order to define the final results.

## Results

We explored professionals’ perspectives on the support of and collaboration with carers with a migration background. The analysis shows that professional perspectives are complex and layered and that underlying social categories influence their daily practice. Four main themes are identified: (1) ‘*The difficult Other*’ which refers to professionals’ perspective on the difficulties they encounter within care networks which they attribute to the ‘Other’; (2) ‘*The dependent Other*’, showing professionals’ understanding of difficulties as intensified by the care context, resulting in a state of dependency of the Other; (3) ‘The uncomfortable self*’,* with professionals describing how insecurities evoked by the Other lead to an inability to act ‘professionally’, and; (4) ‘The reflexive self*’* with professionals reflecting on how their personal and professional identities were implied in complex and diverse care networks.

A Self-Other binary becomes visible within the results. In the interviews, we identified a ‘Self’ presented by professionals’ either with or without a background of migration and an ‘Other’ which, according to the participating professionals, represent carers with a migration background and occasionally also the care recipients. The first three themes are based on the stories of Dutch professionals belonging to the white majority. Within these themes it can be seen that the intersection of being Dutch/white and professional becomes problematic when white privilege is not noticed or confronted and opportunity arises to attribute the underlying reasons to the other. These themes focus on the uncovered aspects of social injustice and/or underlying power inequities within the collaboration. Within the fourth theme relevant intersecting social categories identified by participants themselves become visible and are therefore only explicitly mentioned within this theme.

### The Difficult Other

When asked about their experiences in collaboration with carers with a migration background, participants presented most carers with a background of migration they come in contact with as ‘complex’ or ‘difficult’ based on ‘their’ cultural background and identified them as ‘the Other’. During the informal conversations professionals recognized great willingness to provide informal care which professionals attributed to the non-Dutch cultural background of carers. Pre-assumptions of professionals about care attitudes in different cultures became visible; a Dutch policy officer for example describes ‘*with non-western clients that’s the way it is in their culture’* (I2) and a Dutch community nurse explained ‘*there is more a We-culture, family takes care of the support needs’* (I6). Professionals experience this emphasis on family care often as causing difficulties especially within larger families, where it is often unclear to them who is the actual carer providing care and who is the spokesperson. A Dutch policy officer explains that within her care organization, ‘*it is difficult, especially in Muslim families that there is one person appointed as primary carer who is the contact person with the professionals, and not necessarily the person providing the actual care. Often the father, brother or uncles are the primary spokes persons and the daughters-in law the actual carers’* (I3)*.*

When the division of responsibilities within families is unclear to professionals, miscommunication between professionals and carers may occur, in particular when family members have diverging ideas on caring responsibilities. Professionals attribute this to different levels of acculturation in the Netherlands society. For example a Dutch social worker finds ‘*dealing with the struggles within the family stressful when carers are not accustomed to the Dutch norms yet’* (I16). Additionally, conflicts between professionals and carers emerged when gender roles within families skewed the division of care responsibilities within families. A Dutch occupational therapist describes an example of a Somalian carer who suffered severe carer burden due to lack of care division of care within the family: *‘She has a big family. Her nephews and uncle came by every week to visit the nursing home, but they did nothing. They are not allowed to* [based on their cultural background]*. Only women from the family are allowed to help’ (I11).*Such care division is experienced as difficult when this does not align with professionals own personal values. It is not the particular situation or family, but the generalized culture that is perceive as problematic in these cases.

Furthermore, participants assume that the intersection of ABI with a minoritized cultural background creates a particular vulnerable position for carers in society. Several participants explain how families cope with the invisible consequences of ABI, which proved difficult in some cultural groups. A Dutch care organization director described that ‘*in some cultural communities there is a lack of knowledge and understanding*’(I17). This lack of knowledge and understanding may results in a lack of acceptance by the community, which in turn leads to social isolation of carers. The social isolation of carers is is exacerbated by carers not reaching out to healthcare professionals out of loyalty to the care-recipient. A Dutch occupational therapists experienced that, ‘[They] *do not want to ask for help as they feel ashamed they are unable to provide the care themselves*’ (I12), as there may be a cultural obligation to take care of your family. A community nurse notices that ‘*they* [carer with a background of migration] *never reach out*. *In all those years working in primary care I only had one carer who came* [after discharge] *out of her own will to ask for help’ (I6).* Again, the cultural difference of carers is perceived as the underlying problem for health professionals to offer support in the home context. On top of that, difficulties are linked to unrealistic expectations of carers towards professionals and care organizations: ‘*They demand too much*’ (I8). Consequently, these ‘demands’ result sometimes in irritation and feeling annoyed towards carers. A social worker gives an example: ‘T*hey expect that we do everything for them*, *that is just not the way it works here*’ (I16). This mismatch impacts the relationship between the professionals and the carers as many participants feel a lack of appreciation by carers and care recipients.

### The Dependent Other

In the Dutch context of care, a norm of self-reliance and person-centered care exists that assumes that everyone is capable of self-reliance or active control over their care situation. In our informal conversations, professionals recognized that this ‘difficult Other’ does not readily fit within this existing norm, and that this mismatch may create a ‘dependent Other’. Professionals attributed this dependency to health illiteracy of carers of certain cultural groups’ and to carers’ care attitudes which make it difficult for professionals to adequately support the carers. A Dutch community nurse explains: ‘*They just don’t know the way in the swamp of different laws and regulations which are carried out by different organizations’ (I6).* The bureaucracy and lack of knowledge to navigate the system renders carers dependent on professionals who invest extra time or on knowledge in their own social network. Knowing the way is based on coincidentally having a professional within your care network who bridges the gap to the home context, often this is not the care which has a worrisome effect that carers are often invisible with the Dutch healthcare system.

Especially after discharge carers become invisible within the Dutch healthcare system, A Dutch occupational therapist explains that this is worrisome ‘*because at home the confrontation with the limitations of an ABI become visible and it hits that the situation is vulnerable and there are many struggles, so that’s the moment carers reach-out, but not carers with a background of migration’(I12)*. Some participants realize that that non-western carers are dependent on the possibilities of the healthcare system. They express feeling friction as they realize how carers do not fit the rigid and standardized way of working. Nevertheless, responsibility is however placed on the other as participants argue that carers with a background of migration have learned to be helpless. A Dutch informal care policy officer talked about the responsibility of carers and how this contradicts with her own views on care responsibilities and self-control: ‘*I noticed that a lot of non-western carers have learned to be helpless. I believe that we are in full control of our actions. Your decision-making skills should not be influenced by your faith or any culture, only then can you cultivate your own values and beliefs. I feel strongly that everyone has the same chances in life and that you paddle your own canoe. I try to encourage carers to do so but it does not always pan out the way I've hoped’* (I1)*.* Putting emphasis on self-reliance, which is a deeply Western individualized cultural belief, placing the ‘blame’ of not fitting in completely on the shoulders of the dependent ‘Other’ is overlooking the unequal position of carers with a background of migration and not recognizing the need for rapprochement within care networks as a responsibility of everyone, contributes to inadequate collaboration within care networks.

During the interviews participants expressed their struggle with the fact that the dependent other does not readily fit within the system. Standardization of care interventions form a juxtaposition with the need for person-centered care in which the uniqueness of each person is seen as essential. This is an impossible situation based on previous observations that carers often do not know their way in the health care system. Additionally participants describe that carers also not have equal opportunities to access healthcare. Professionals recognize that carers and by proxy care recipients may not have the means to access healthcare after an ABI. For example, participants mention that the lack of supplementary insurance is a struggle for carers with a background of migration: ‘*They do not have the financial means for this kind of insurance’*. But without this form of insurance additional but necessary care needs to be paid out of pocket, and this is often not possible, which again leads to skewed access to healthcare services.

The dependent other also becomes visible in the lack of access to professional translators within the healthcare system. As participants mention language as the main barriers within the collaboration, a Dutch-Surinamese creative therapist tells that: *‘When I think of non-western clients I think of the language barrier, in addition to the language barriers from the ABI such as aphasia*’ (I12). There is a large need to have contact with carers who speak Dutch or work with a translator, placing responsibility for unequal access to care on carers as a translator is ‘*not always readily available’ (I7)*. However, participants also express that there is a gap in health care: ‘I*t’s not okay when people do not understand you!* or ‘*nobody talks about their fears, anger or sadness in a second languages’ (I16).*

Struggles between professionals and carers also emerge when family norms and values of caring for someone interfere with care provision. A Dutch occupational therapist explains, *‘I always put a large emphasis on the self-reliance of the care recipient, with the aim of not losing more function after the brain injury than is already lost’* (I7). This focus often leads to debates between professionals and families as families tend to take over all activities, keeping the care recipient dependent of the carer. A Dutch social worker provides an example where care recipient expectations did not fit within Dutch Social Support Act: ‘*A woman from Egypt only has her son here in the Netherlands. Due to incontinence as a consequence of her ABI, she has severe bedsores and is unable to take care of her house. She explicitly does not want her son to take care of the house because he is a man’ (I16).* This mismatch impacts the relationship between professionals and carers as such a statement by care receivers may cause professionals to experience a lack of appreciation by carers and care recipients.

Dependency is seen in the role of the carer themselves, as they do not have a formal place in the care network. Carers depend on professionals, organizations or policy to assign them a place in the care network. *‘*Sometimes participants ask questions to the carer, ‘*how are you holding up, do you manage with work or do you also focus on yourself?’ (I10).* But in many organizations participants are not required to discuss care or choices with a carer, ‘*this is the responsibility of the care-recipient’* or ‘*they need to give consent to talk to the carer, which proves difficult sometimes with an ABI (I6).* For the interviewed professionals, this was stressful as they felt caught in the middle when trying to meet the expectations of the care recipient and carers. Care was constrained by the possibilities of the care context.

### The Uncomfortable Self

Collaborating with a carer with a non-majority cultural background may lead to feelings of insecurity and stress. A Dutch occupational therapist explains ‘*I have stress when I do not know what to do, do I need to take of my shoes? May I refuse more coffee? When I am with a Dutch family I ask, but here I do not’* (I13). Unfamiliarity with gender roles in different cultures makes participants uncertain about their possibilities as a professional, a Dutch community nurse askes: ‘*Are we allowed to wash a man when only his wife is allowed to see him naked?’* (I6). Insecurities are also observed in the discomfort surrounding terminology when talking about carers with a migration background, such as ‘foreigners’, ‘immigrants’, ‘westerners versus non-westerners’, ‘them and they’, pointing towards a lacking vocabulary for speaking about diversity issues. Professionals’ discomfort could be literally observed during the interviews in actual physically retreating bodily posture when discussing the matter, or accompanying it with laughter.

Uncertainties might stem from the topic’s political sensitivity, a fear of doing or saying something wrong, or even be viewed as a racist. Racism or discrimination was mentioned by two participants who wanted to make abundantly clear that they themselves were not discriminating. A Dutch occupational therapist expressed that cultural differences are a struggle for her for example when carers present as victims and lack self-reliance and place their locus of control outside themselves. She described her feeling, ‘*dammit again a Moroccan who believes that she is a victim…I do not want to deal with that anymore’* (I12). Another extreme example was given by a Dutch social worker, who described a complex situation in dealing with a care recipient who called her a racist, after which she called the police department to report him: ‘*Dutch people never say they get discriminated against, this always concerns someone with a different culture. I feel that this is learned behavior and I believe that when you are firm and do not tolerate being called a racist this will not happen again’* (I16). Both professionals expressed a justification for their comments with a reflection that this is not a discriminatory way of thinking.

When speaking about lack of self-confidence, a shift in the professionals’ reasoning becomes visible in their stories. All participants comment on the role of diversity, and argue that carers are the same, to justify their earlier comments on the difficult other. For example a Dutch occupational therapist argues; ‘*occupational therapy is aimed at the individual and has therefore nothing to do with diversity*’ (I11) or a director of a care organization explains his worldview ‘*person is a person to me no matter if they look different’ (I17).* With this sudden turn, professionals completely negate the role that aspects of diversity play when their reflections, assumptions and predefined expectations could not be reconciled with their professional identity.

### The Reflexive Self

Some participants reflect on their personal and professional identities and how these relate to their work. In general, collaboration with carers with a migration background is perceived by some participants as fun or a learning opportunity. A policy officer likes to submerge herself in different cultures: ‘*I like to learn from different culture’* (I2). Some participants explain how cultural differences are a struggle for them, for example when carers take on what they perceive as ‘an attitude of victimhood’. Thereby several participants reflected on their own role with differences in care networks, and recognized the need to be open minded and the necessity to ask questions and to not make quick assumptions. A Dutch occupational therapist reflected: ‘*I realize that I have blind spots… I need to make contact first before going further in the process’* (I13)*.* Often, participants realize that there is a need to let go of the expert opinion and first, learn from different cultures. A Dutch Afghan social worker recognizes the need to be open minded and not assume to quickly: ‘K*now that knowledge is power but that you do not know everything even though you are the professional’* (I15).

Subsequently, some participants identified within their own identity, two intersecting aspects of diversity as relevant regarding the professional perspective: being an educated professional on the one hand, and seniority within the workforce or having a migration background themselves on the other hand. Senior professionals sometimes recognized that reflection is required on the validity of their knowledge. This Dutch-Peruvian community nurse explained how she falls back on her work experience when trying to work in a partnership with carers: ‘*Throughout the years as a nurse I have learned to perceive people as my partner, being the professional who thinks they know best will do more harm than good in my opinion’* (I5). A Dutch-Turkish occupational therapist who for years works in the same culturally diverse community adds to this by explaining that she does not have a manual to fit every person. To her, person-centered care has added value and she explicitly mentions the autonomy, or power of doing things differently every time.

Some participants mention how having a migration background as a professional was helpful in itself when supporting carers. A Dutch-Peruvian community nurse explains: ‘*I am from Peru myself and I speak several languages. So I am able to communicate with them on their level’* (I4). They found it easy to build trust, which is necessary for carers to be open about their situation and support needs. This is not always the case. A Dutch-Afghan therapist explains that to her, sharing an assumed similar background with care recipients can also be burdensome: ‘*I often go on home visits where people have the same cultural background as me. They put an extreme pressure on me by saying things like… ‘I am so happy that you understand me completely’… while I have no idea’*(I15). The participant is annoyed having to explain over and over again that she does not understand everything, and this has a major impact on work pressure and pleasure. Thus, although professionals’ migration backgrounds may be an added value, they are not automatically so. And finally, although people may share group memberships, stereotypical assumptions and expectations can also be applied to professionals by care recipients and carers.

## Discussion

This study gains insight in diversity in care networks by exploring professionals’ experiences in collaboration with informal carers with a migration background. We will now discuss how the four themes help to understand the complex reality of this collaboration, which is influenced by professional and personal identities and the context in which care is provided. We will also argue that our intersectional analysis offers an explanation for the health inequalities found by other scholars who studied diverse networks and discuss what is needed to overcome these inequalities.

Our intersectional analysis reveals a dynamic process of feeling, thinking and acting in professionals’ description and understanding of their collaboration with informal carers with a migration background. Professionals’ experiences are influenced by aspects of diversity of both the diverse Other as well as the diverse self, which is captured in the four themes: *(1)’The difficult Other’, (2)’The dependent Other, (3)’The uncomfortable self’* and *(4) ‘*The reflexive self*’.* The four themes are interrelated, and can be seen as part of what Brons [14] denotes as a dialectic process in which Othering is the ‘simultaneous construction of the self and the other in mutual and unequal opposition through identification of (un)desirable characteristics. This opposition sets up a superior Self in contrast to an inferior other, but that is nearly always left implicit.’ (p.70).

The process of Othering becomes explicit and visible when professionals identify the other as (1) difficult when problems arise within the collaborative network, and (2) dependent when they do not fit within the structures and norms of the Dutch healthcare system. This identification as the difficult other is based on intersecting aspects of diversity such as culture, family systems, and gender roles, and associated stereotypical assumptions. These assumptions are often taken-for-granted and highly unconscious among professionals, and so are their normative persuasions of how carers and their care recipients should behave according to the dominant societal norms and policy regimes. In such a situation it is hard to build a relationship and attend carefully to the specific needs of the carer and the particulars of the situation. This is in line with Alpers [8] who argues, from a care recipient perspective, that Othering might lead to distrust within care relations, and a recent study of Claeys et al. (2021) who show that professionals are ‘likely to portray their own frame of reference and find it challenging to show empathy with patients with a different background’ (p. 484). Moreover, there is the risk that professionals from their perceived superiority will use their expert power to determine what is needed by whom. If professionals only see the generalized other they may not assess and offer what is needed by the particular informal carer(s) in a diverse care network, which may lead to health inequalities. In this study, we see how the process of Othering offers an explanation of these undesirable outcomes, and that Othering needs to be addressed to avoid health inequities and related negative influences on health outcomes.

Misunderstandings and conflicts within care situations become visible, within the ‘Dependent Other’ as professionals narrate about their struggles in the collaboration that derive from this dependency. Tensions and conflicts arise when carers with a migration background do not readily fit within the Dutch healthcare system, which is set by neoliberal health and welfare policy. This finding is in line with Peacock et al. [42] who argue that the experienced dependency can be the result of internalized neo liberal discourse. Neo-liberal policies have an aggravating effect of the ‘access to healthcare services for groups that are already experiencing difficulties’ [46], p. 7). Othering is then often used to serve to ‘both position the self in a safe and defensible space and to endeavor to make sense of what is felt to have changed in the wider world’ [42]. The dependent Other is a construction in opposition to and harnesses the independent professional as neutral Self, and this Self-Other binary prevents a mutual partnership between professionals and informal carers.

Subsequently, insecurities and stress are observed amongst professionals within the collaboration with carers with a migration background which is captured in the ‘Uncomfortable Self’. Prejudices of professionals about other cultures and habits of carers create tensions and barriers within the collaboration. This also works the other way around, when informal carers have different expectations of the provided care, which may lead to distrust fueled by preconceptions of professionals [8]. Within the ‘Uncomfortable Self’ it is recognized that professionals do not question situations that are unknown to them as they have the feeling they are required ‘to know as the expert’ professional. This is also observed by Claeys et al. (2021) who identify fear amongst professionals to be seen as racist, which prevents them to ‘communicate and act in a spontaneous way’ (p. 4). Instead of defining oneself as all-knowing and superior to the other, professionals may redefine their identity to develop a more caring and fruitful relationship with informal carers [2]. The Self-Other binary can only be deconstructed by reframing the identity of the professional and the relationship as being deeply interdependent and interconnected. Only, then a partnership approach can start to develop among professionals and carers in a diverse network.

The ‘Reflexive Self’ describes professionals who reflect on their own identity and its impact on their daily practice. Reflexivity towards the role of diversity and participants’ own identity shows a level of sensitivity necessary to overcome social injustice recognized in the participants experiences. Namely, being sensitive towards carers allows them to speak their mind, recognize multiple truths within a care network and create a space where the other is also heard especially those who are not typically acknowledged and heard as an ‘expert’ in their role. This requires that all stakeholders in care networks, at personal, professional and organizational levels, enter into a multicultural dialogue [28]. The lack of reflexivity may reinforce unchallenged assumptions about the Other [32]. Professionals often do not recognize the need for help as they simply do not recognize the signs that an informal carer is in need, or they lack knowledge about the cultural background of the informal carer. In order to widen access to healthcare, there is a need to be sensitive for blind spots, based on personal identity and social position. Letting go of the illusion of being all-knowing and that all tensions can be technically solved, the reflexive practitioner starts from a more humble attitude, acknowledging the wisdom of informal carers and their longstanding relationship with the client [5]. Changing how professionals communicate can alter power dynamics and reconfigure healthcare practices.

## Strengths and Limitations

The use of intersectionality as an analytical framework in the secondary analysis is a strength in this work showing the implicit influence of contextual factors and intersecting aspects of diversity becomes explicit [50].

As seen in the results of this study the original themes are colored by a deeper, otherwise invisible layer, of diversity and power. Additionally, the data collection and analysis of the dataset consists of 17 interviews, 12 informal conversations within health and welfare organizations, a CoP and two critical friends in order to create a strong sense of data- and researcher triangulation. Although some participants had little contact with carers with a migration background data saturation was still met. One of the limitations of this study is the fact that some of the included participants had limited contact in their work with carers with a migration background.

## Implications

From the professional perspectives it becomes evident that an interrelated process of Othering, including the construction of a self-other binary, plays a role in diverse care networks, which offers an explanation for health inequalities that have been evidenced. From the professional’ perspective the collaboration in these networks is loaded with tensions and misunderstandings. It would be beneficial to focus further research on the perspectives of carers with a migration background and care recipients and uncover their experiences in the collaboration within care networks.

An intersectional approach can be helpful to understand the self-other binary that appears within collaborative care networks surrounding care recipients with ABI. The rather essentialist perspective of professionals on carers with a background of migration may be broadened when an intersectional perspective is introduced. Such a perspective may help professionals to gain knowledge and insight in differences and inequalities within and between groups of carers they collaborate with, now often in general referred to as ‘them’ or using broader overarching terms. Additionally, an intersectional perspective may provide insight in the intersections of diversity categories that are embedded in a specific context of care networks. This may help to highlight and understand the diversity amongst carers with a migration background. Professionals may then be able to move beyond an essentialized view on ‘cultural difference’ when working with carers and by proxy care recipients with a background of migration.

Within educational programs and within practices there is a need to explore how reflexivity can become an essential competence of what it is to be a professional to ensure equal access to healthcare. ‘Reflexivity can help transform the collaboration when people involved bring critical self-awareness, role-awareness, interrogation of power and privilege, and the questioning of assumptions and ‘truths’ to their work’ [27, 28], p.10). Reflexivity is the process of engaging in self-reflection about who we are as professionals and how subjectivities and biases guide and inform daily practices [34]. Reflexivity requires one to enter into what Donald Schon [47] calls a ‘swampy lowland’, that is the uncomfortable place where technical solutions do not work, because of uncertainties and ambiguities and where one is involved on a personal level. Here the comfortable shield of professional neutrality can no longer be upheld, which often causes emotional pain, moral uncertainty and existential unrest. No need to say professionals rather avoid such a place, and often have the privilege to do so because they do not regularly have to reflect on their cultural background as they are not confronted as being different [21, 52].

Therefore the integration of reflexive practices in healthcare curricula is needed as diversity responsive care requires a critical investigation of oneself as professional. This reverses the traditional way in which the self-Other binary is approached [35]. Instead of studying the other, the Self becomes object of scrutiny.

## Conclusion

The collaboration between professionals and informal carers with a migration background is subject to and reinforces underlying power structures and aspects of diversity. A process of Othering and Self-Other binary becomes visible in unequal relationships and tensions within the collaboration. Amongst participants, levels of insecurity emerged when a variation of action was needed, which did not belong to the core competencies of their profession, which fueled the feeling of not being an adequate professional. Being reflexive as a professional and letting go of the illusion of being all-knowing and in control of confusing situations will allow for the identification of blind spots and power asymmetries within the collaboration. We would therefore call upon educational curricula and professionals to invest in the integration of reflexive practices through participatory action research, unsettling the Self-Other binary by a critical scrutiny of oneself as part of an interconnected and interdependent care network.
